# Influenza*Read before the Veterinary Medical Association of New Jersey.

**Published:** 1894-01

**Authors:** W. B. E. Miller


### INFLUENZA.*

By Dr. W. B. E. Miller

Influenza, also known as epidemic bronchitis, catarrhal fever, shipping colds, distemper, etc., but more commonly called pink eye, is a blood disease, with both local and constitutional expressions, which may assume different phases, dependent on sundry influences operating in various ways, generally epizootic, with a tendency to spread. It is also a panzootic disease, arising in different parts of the earth simultaneously, and in this respect is similar to cerebro-spinal-meningitis. When the atmospheric, or other influences producing the blood poison, extends over a large area it assumes an epizootic form. When these conditions are limited and the poisons emanate from purely local surroundings or from the soil, it is called enzootic ; while isolated cases appearing here and there, causing no infection of other animals, are termed sporadic.

The season of the year is supposed to have some influence in its production, it being, as a rule, more prevalent during the spring and fall months. But it matters not in what form it may appear, or at what season of the year, the principal symptoms and lesions are one and the same in all cases, and are readily recognized by those familiar with them.

It is characterized by an early elevation of temperature, not unusually changing from that of normal to 104 to 107 degrees F., or even higher within twenty-four hours. The pulse is usually feeble, soft and quick, but not frequent. The animal will have a dejected appearance in the face. There may be an early chill, followed by sudden and marked debility. The head droops, the muscles relax, and the animal becomes very weak, sometimes crossing his limbs, or if forced to move will reel or stagger as he walks. There are also elementary symptoms which, in my experience, are especially characteristic, namely : tracheal bronchial, or pulmonary catarrh, and continued fever, either of which may predominate, but usually the latter. The animal appears to be suffering from pains in the head (headache), partial deafness from the ringing in the ears, dimness of vision from swelling of the eyelids and the membrane nictitans, pains in the nasal sinuses from swelling of the mucous

* Read before the Veterinary Medical Association of New Jersey.

membrane lining the same. The animal rapidly loses strength without losing any flesh.

In some cases early symptoms may be mistaken for spasmodic colic. The horse will paw, kick, get up and down frequently, but gently, not throwing himself down as if really suffering from colic. These symptoms are of short duration, and the animal soon assumes the upright position, but with drooping head and dejected countenance. A low and debilitating fever (typhoid in character), with external muscular debility, very soon follows. The schneiderian membrane, the membrana-nictitans, and other visible membranes, become greatly injected to a great extent, presenting a pinkish red color, which probably gave rise to the name of "Pink Eye." The eyelids become swollen, sometimes to an alarming extent, often being entirely closed. A watery secretion and often pus is discharged from them, and from the nasal passages, a catarrhal discharge excoriating in character. The smell is impaired, the throat is sore. The cough, when present, as it generally is, is harassing.

Exploration of the chest reveals nothing uncommon, unless pleurisy or pneumonia complicate the case, and when present, they are usually of a "typhoid type," and are not accompanied by some of their marked symptoms ; for instance, the severe pain of pleurisy, or the nasty brick dust sputum of pneumonia may be entirely absent. Capillary bronchitis may also intervene. These three conditions are not as a rule found in the early stages of the disease, but more often are secondary, following as a sequence, rather than being complications.

The digestive organs may also be affected, and diarrhœa be present. If so, and when of not too long duration, it may be beneficial to assist nature in eliminating the poison from the system The urine is scanty, turbid, and heavy with urates, and is voided with some difficulty, which may be due to the fact that the animal finds it difficult to assume the proper position for the act, owing to the weakness and soreness of the muscles.

A horse suffering from influenza seldom, if ever, lies down, not because of embarrassment to respiration, as is pneumonia or pleuro-pneumonia, but because of the general soreness aud stiffness of the muscles and of the extreme debility. The animal apparently knows that if he gets down he will be unable to rise again. He may become so weakened that he will fall down, but as a rule he will not do so voluntarily. When he does so, it is a very favorable symptom. Sometimes the disease is complicated by

"epistaxis," and also assumes a marked typhoid character, or it may take on a rheumatic or erysipelatous form, or it may assume a dropsical character and terminate in "purpura hæmorrhagica" with œdema of the limbs, abdominal and pectoral regions.

One form of the disease (the pharyngeal) manifests itself locally, with but slight cough and soreness on pressure, yet the animal has great difficulty in swallowing. If water is given him he will make an effort to drink, but fails to swallow much, if any, the water returning through the nostrils and thence back into the bucket. In this respect this disease is similar to "Cerebro-Spinal Meningitis," as we have this symptom in that disease but with this difference, that in the latter the animal cannot trough his tongue owing to the affection of the nerves controlling that organ, and although he may make every effort and plunge his head into the water up to his eyes, he cannot draw in the fluid, and when the bucket is removed it runs out of the mouth instead of through the nostrils as in the former case. Another peculiarity of this symptom of inability to swallow, is that he becomes unable to swallow fluids sooner than he does solids, that is, he can continue to swallow solid food after he is unable to swallow water. In my opinion and experience the pharyngeal form of the disease is more liable to assume a malignant type, accompanied by a fœtid odor, and later on by an offensive discharge and sloughing, resulting in septicæmia and terminating in death, due to the fact that the poison had all been absorbed into the system during the early stages of the attack, instead of having been eliminated therefrom by early discharges, as in the catarrhal or other forms of the disease. In severe cases of purpura hæmorrhagica, with extensive swelling of the limbs and abdominal and pectoral regions, wherein the poisonous material has become so thoroughly absorbed that it cannot be eliminated, or its action overcome for some length of time, the same condition of sloughing is likely to appear in the swollen parts, when not only the skin, but the intercellular tissue, and even portions of the adjacent muscles, become involved in the process of decay. The sloughing goes on rapidly in these cases until death or the poll axe puts an end to the suffering of the poor brute. In influenza in whatever form it may appear, or however much it may be complicated, the marked muscular debility is always present and manifested primarily ; while in severe cases of other diseases of the throat, bronchial or lungs, after days of sickness from depletive treatment, or from lack of proper nourishment, it may and often does ensue, but is always a secondary symptom and the sequence of other conditions. The course of the disease is, as a rule, regular, convalescing in from seven to fifteen days, and ends as do nearly all blood diseases, in leaving the patient very much enfeebled, and often with swollen limbs, which may remain for some time after the patient has to all appearances otherwise entirely recovered. With proper treatment, and when uncomplicated, the rate of mortality should not exceed 1½ to 3 per cent., according to surroundings. Good care, warm quarters (well ventilated), and nourishing food, in moderate quantities, have much influence on these patients, and when associated with good treatment, are generally succeessful in bringing about a good recovery.

No class or particular breed of horses is exempt from influenza, yet I am of the opinion that the more heavy animals, draft horses and others plethoric in character and in full flesh, are more susceptible, and seem to become more seriously affected by it. Some authors and practitioners claim that horses owned and kept in the country districts do not appear to be as good subjects as those kept in cities, they being more exposed and kept more in accordance with the state of nature. My own experience, however, leads me to place considerable doubt on this statement. Animals recently brought into our locality from the western sections are generally attacked soon after their arrival, or in course of shipment, hence the term "Shipping Colds," as used by the dealers. Young animals seem more susceptible than older ones, but the older ones seem to have the disease more severely, and are less able to endure the suffering and weakness consequent upon the attack. When they do take it, it seems to have a more exhausting and enfeebling effect upon them in every way.

Many cases that I have seen in old horses were a long time convalescing, and not a few failed to make a complete recovery, being left with some other disease or dying from the disease itself.

The lesions as seen in post-mortem examinations depend very much on the local manifestations of the disease during the attack. The heart is usually placid, containing fluid blood or a soft post-mortem clot. Bladder is empty or nearly so, and shows slight inflammatory conditions of the membrane. The urine, if any, is dark colored and turbid and heavy with urates. Kidneys are slightly congested and softened. The intestinal is tract, apparently normal, unless typhoid symptoms have been present, when Peyer's patches will be found to demonstrate that fact. If the catarrhal symptoms were extensive you may find excoriations of the nasal sinuses and the air passages with probable sloughing of the same, or perhaps the lesions of a passive pneumonia. Most generally, however, the principal lesions found are those of a bronchitis or catarrh, or both.

### IS THIS DISEASE CONTAGIOUS?

Some authors, Gamgee and others, claim that it is. Others, that it is not. I am inclined to the latter opinion, no positive evidence having proven to me its communicability from one animal to another. Certain it is that it is infectious, but the active principle that produces the poison or carries it from place to place, and from stable to stable, and infects one animal, may also infect ten or even a hundred if they come in contact with the poison; and yet no one diseased animal is capable of reproducing the disease in another when the active principle or poison is absent.

### CAUSATION.

Many theories have been advanced as to the cause of this disease. Some claim that it is due to ozone in the atmosphere; others, that it is due to a microscopic animalcule; others, that it is due to a change of weather, as a sudden change from wet to dry, or from heat to cold; others, that it is due to injurious noxious gases. Various other causes have been assigned, but all, in my opinion, lack positive proof. Certain it is, that whatever may be the cause, whether exciting or predisposing, or both, it is a material one, and well worthy of more thorough investigation than the profession have thus far given to it, either scientifically or practically.

A disease so prevalent as this has been for several years past, really demands more scientific study. There is no doubt but that the conditions of the atmosphere, and certain bad conditions of surroundings, seem to excite a powerful influence on animals when sick. whether they have any effect on the production of the disease or not. This is also true in relation to the season of the year when the attack takes place.

### TREATMENT.

The least meddlesome the better. Perfect rest, good warm stables, warm clothing, warm vegetable and mucilaginous drinks are necessary.

Diaphoretics are indicated, but your patient becomes weak so rapidly that they should be given early and carefully. Do not deplete in any way if you can avoid it, as the tendency of the disease is to exhaustion. Good fresh air, without any draft over the patient, is very essential. The general treatment early indicated is stimulative, something to keep up the strength.

The low debilitating fever is here as an essential part of the disease, and your patient will take no food at all at this stage of the case, and care must be taken therefore to preserve the strength and subdue the fever at the same time. Bleeding is not allowable, because your patient is already becoming enfeebled. You must therefore use febrifuges in connection with stimulants. Spirit Nit. Dulc. and Opium in strengthening doses are always indicated in all pulmonary diseases, and are very useful in this.

Purgatives are inadmissible. Laxative food is all that is required to keep the bowels regular and in good condition. Give stimulants and tonics—quinine, cinchona, ammonia and alcoholic stimulants All are indicated, and are in the line of good treatment if properly administered If the rheumatic symptoms of the disease are found, give salicylic acid, or salicylate of soda in conjunction with nitrate of potash, carbonate of ammonia, etc. Emulucent drinks made of flax-seed tea, mucilage, licorice root, etc , are sometimes beneficial when the cough is distressing. Any treatment that has a tendency to allay the fever and strengthen the patient is admissible. but good ventilation in the stable, without a draft over the patient, good warm clothing, perfect rest, warm drinks, warm nourishing food and good care are essentials. If symptoms or complications, such as pneumonia, rheumatism, excessive dropsical effusions or swelling of the limbs, or purpura hemorrhagica should arise, you must act accordingly, and treat the symptoms as soon as they appear, so as to cut short, if possible, the condition presented.

As influenza and cerebro-spinal meningitis usually occur at the same seasons of the year, it is not unlikely that you may have the two in the same stable at the same time, each of which might be at first mistaken for the other. In fact, I have seen cases wherein, I think, I had both diseases at the same time in the same animal. Here you will have to be guided altogether by your temperature. In the former you have it ranging from 104 to 108 deg. F., with great muscular weakness all over the body; in the latter you have it from 99 to 102 deg. F., never reaching 104 until near the close of life, unless you have influenza with it.

In the latter you also have the peculiar dragging of the toes of the hind extremities, and loss of the power of deglutition, from paralysis of the tongue. If both conditions are present, you will have a staggering, dragging gait, combined with dragging the toes, and a temperature of 103 to 105, and variable with other well-marked symptoms of both diseases.

This condition would naturally be thought to be very dangerous, but I have not found it positively so. One poison seems to antidote the other; the influenza acting as a safeguard against the dangerous inroads made by the other disease. In cases thus complicated, purgatives are indicated and are beneficial in aiding one to eliminate the two poisons from the system quickly. Use stimulative treatment afterward to restore the strength, and tonics to build up the system.

